# Mothers’ Perspectives of Complementary Feeding Practices in an Urban Informal Settlement in Kisumu County, Western Kenya

**DOI:** 10.1093/cdn/nzab065

**Published:** 2021-04-14

**Authors:** Elise C Reynolds, Dickens Onyango, Rael Mwando, Elizabeth Oele, Thomas Misore, Janet Agaya, Peter Otieno, Beth A Tippett Barr, Gwenyth O Lee, Victor Akelo

**Affiliations:** University of Michigan School of Public Health, Ann Arbor, MI, USA; University of California, Davis, Davis, CA, USA; Kisumu County Department of Health, Kisumu, Kenya; Kisumu County Department of Health, Kisumu, Kenya; Kisumu County Department of Health, Kisumu, Kenya; Kenya Medical Research Institute, Kisumu, Kenya; Kenya Medical Research Institute, Kisumu, Kenya; Kenya Medical Research Institute, Kisumu, Kenya; US Centers for Disease Control and Prevention–Kenya, Kisumu and Nairobi , Kenya; University of Michigan School of Public Health, Ann Arbor, MI, USA; US Centers for Disease Control and Prevention–Kenya, Kisumu and Nairobi , Kenya

**Keywords:** infant, child, mothers, poverty areas, informal settlement, undernutrition, Kenya, urban, complementary feeding

## Abstract

**Background:**

In informal settlements, the benefits of urban dwelling are diminished by conditions of poverty that exacerbate child undernutrition. The Child Health and Mortality Prevention Surveillance (CHAMPS) project has identified malnutrition as the leading underlying cause of death in children under 5 in the Manyatta urban informal settlement in Kisumu County, Kenya.

**Objective:**

This qualitative study, nested within the CHAMPS project, aimed to understand community perspectives on complementary feeding practices in this settlement.

**Methods:**

In-depth interviews were conducted with 20 mothers who lived in the urban informal settlement and had a child 6–23 months old. Two focus group discussions were conducted, 1 with mothers and 1 with community health workers (CHWs), to further explore themes related to complementary feeding.

**Results:**

Mothers were knowledgeable about globally recommended feeding practices, but such practices were often not implemented due to *1*) the community/household water and sanitation environment, *2*) the community/household food environment, *3*) a lack of income and employment opportunities for women, and *4*) sociocultural factors. Together, these create an environment that is not conducive to optimal child feeding practices.

**Conclusions:**

To improve complementary feeding practices and child nutritional outcomes in Kenya's informal urban settings, both community- and individual-level factors should be addressed. Possible interventions include investment in water infrastructure and social protection programs, such as cash transfers.

## Introduction

Urban dwelling has often been associated with improved health and nutritional status, especially among children under five; however, these benefits are reduced or absent among children living in urban informal settlements, who face high rates of multiple forms of undernutrition, especially stunting (1–6). As the world's urban population grows to reach 68% by 2050, and with greater than 53% of urban sub-Saharan Africans living in informal settlements, the number of children facing these disadvantages is only predicted to grow (7, 8).

In Kenya, over one-third of children in informal settlements in the capital city, Nairobi, are stunted (9–12). In this setting, inappropriate complementary feeding practices, compounded by conditions of poverty, increase the risk of childhood stunting (13, 14). Factors such as a child's sex and age, as well as the mother's employment, education, socioeconomic status, and prenatal health-seeking behavior, have all been related to complementary feeding outcomes (15–17). The neighborhood environment is also closely associated with infant and young child feeding practices, with factors such as poor sanitation and the presence of street vendors enabling poor feeding habits and creating additional barriers to recommended practices (18). In addition, social and cultural beliefs can contribute significantly to inadequate feeding practices, as can influences from close family and friends (19). For instance, studies continue to report common cultural exclusions in children's diets, particularly regarding meat consumption (20–22).

The Kenyan government has prioritized child nutrition through new legislation in the 2010 revision of the Kenyan Constitution, which aims to promote optimal feeding (23, 24). The Baby Friendly Hospital and Baby Friendly Community Initiatives have been introduced and expanded throughout the country (25, 26). In addition, a national guide to complementary feeding for children aged 6–23 mo was developed, which focused on maternal education and addressed barriers and misconceptions related to feeding (25, 27).

While the nutritional status of Kenyan infants and young childen has improved since 2009, the prevalence of stunting is still 18% in Kisumu county, in western Kenya, where this study takes place (28). In the region, only 25% of breastfed children are both consuming foods from more than four food groups and meeting the minimum meal frequency (28). The Child Health and Mortality Prevention Surveillance (CHAMPS) project seeks to identify causes of mortality among children under-five in 7 sites in South Asia and sub-Saharan Africa, including Kenya's Kisumu and Siaya counties’ Health and Demographic Surveillance System (29). Preliminary analyses of CHAMPS data from Manyatta, an informal urban settlement in Kisumu city, identified malnutrition as the leading underlying cause of under-five mortality (30, 31). Following these findings, the Kisumu County Department of Health partnered with CHAMPS to conduct a rapid anthropometric assesment of children aged 0–59 mo in Manyatta. The prevalence of global acute malnutrion was found to be 1.6% while the prevalence of stunting was 10.8% (D Onyango 2019, personal communication). Despite this level of stunting only being of medium concern by WHO prevalence thresholds, given that malnutrition remains a key underlying condition contributing to the high rate of under-five mortality (79/1000 live births) in the community, it is important to understand the factors underlying these rates (31, 32).

In addition, although substantial research has been done to understand drivers of inappropriate feeding practices in Nairobi, less is known about infant and young child feeding practices in other urban informal settlements, particularly in western Kenya (12, 18, 19, 33–35). And, although the root causes of inadequate feeding practices in Kenya have been conceptualized by researchers, the public health community, and members of some communities, less has been done to understand how mothers actualize complementary feeding recommendations and knowledge into practice and the challenges they face (18, 19). A study conducted in 2 Nairobi informal settlements found that two-thirds of participants knew complementary feeding should begin at 6 mo, but 30% reported introducing food before this point, suggesting that maternal nutrition knowledge alone is not sufficient to improve feeding practices (34). Therefore, this study aimed to qualitatively assess how suboptimal complementary feeding practices are conceptualized and contextualized by mothers living in the Manyatta Health and Demographic Surveillance System of Kisumu city, Kenya's third largest city. Specifically, we examined mother and community health worker (CHW) perspectives on the individual-, household-, and community-level factors that interact with mothers’ knowledge to produce continued inadequate feeding.

## Methods

### Study setting and population

Manyatta has a total population of 77,000 (29). It is served by 2 private and 3 public hospitals, including the regional teaching and referral hospital. Community health services are offered by CHWs from the Ministry of Health, each of whom is assigned ∼100 households. Manyatta has 100 villages that are used as census clusters. In 2019, the rapid nutritional assessment was conducted among children aged 0–59 mo in 40 of these 100 clusters (D Onyango 2019, personal communication). Participants who were found to be stunted or undernourished in the rapid nutritional assessment were referred by their CHW to their local clinic for further screening and enrollment in a therapeutic program as necessary. In this study, mothers and CHWs were purposively selected from the same 40 clusters that participated in the rapid nutritional assessment.

### Data collection

In-depth interviews and focus group discussions were conducted to explore mothers’ current nutritional knowledge, barriers to action, attitudes and beliefs about feeding practices, and sources of information. To sample participants, a cluster was randomly selected from the 40 described above. The CHW from that cluster was contacted by the study team, briefed on the study, and asked to contact a mother in their cluster who had experiences relevant to the purpose of the study, had at least 1 child aged 6–23 mo, and had begun complementary feeding. No mother who was approached refused participation. Random selection of clusters and selection of a single participant within the cluster continued until no new themes emerged and saturation was reached, after 20 fully completed interviews.

In interviews, mothers were asked about their own behaviors, including current complementary feeding practices, challenges they face and how they overcome them, and successes. In focus groups, mothers were asked to reflect on such topics at a community level and describe community success and challenges in complementary feeding. CHWs similarly were asked to reflect on the practices, challenges, and successes that they and the mothers in their community experience (**Table 1**).

**TABLE 1:** Representative questions from in-depth interviews and focus group discussions demonstrating themes that were probed during sessions

**Questions**
**Representative interview questions**
Is there any way in which you wish you could feed your child differently than you actually do? If yes, how so?
What factors do you consider when trying to make a decision around your child and their food?
What challenges or barriers do you face in accessing the kinds of resources you need or want?
**Representative focus group discussion questions**
*Mothers*
Who in the household generally makes the decisions around what children are fed and what food is purchased?
What are some challenges mothers face when feeding their children?
What role does hygiene play in infant and child feeding in the community?
*Community health workers*
What kinds of topics/questions related to nutrition and infant feeding are most often asked to you as community health workers?
What are some of the feeding practices you see in your community that support or do not support child nutrition and development?
What do you think are the biggest challenges your community faces with infant and young child feeding?

Interviews were conducted in English, Kiswahili, or Dholuo depending on the preference of the interviewee. The interviews were conducted by 2 field interviewers, both of whom were fluent in all 3 languages and held a bachelor's degree in nutrition. Interviews were conducted in the participants’ homes. Interviewers participated in a 1-day training to become acquainted with the purpose of the research project and qualitative interviewing skills. Both team members conducted a supervised practice interview and were provided with feedback.

Two focus group discussions were also conducted, 1 with mothers living in the Manyatta settlement and 1 with CHWs serving the area. Each focus group had 11 purposively selected participants. CHWs helped the research team select mothers, in the same manner as for the interviews, for participation in the focus group discussion. Eleven CHWs were selected for focus groups by the research team in collaboration with government officials, community health focal persons, and other community workers. Focus group discussions were conducted at a central location during the lunch hour, to accommodate participants’ schedules. Focus group discussions were conducted by a moderator with extensive experience and a background in social-behavioral science. The moderator met with the research team prior to the discussions to review the discussion guide, and 2 field workers acted as notetakers. Each focus group discussion lasted approximately 1 hour. The language of the discussion was decided by consensus of the group and both were conducted in Dholuo.

In-depth interviews and focus group discussions were audio-taped, transcribed verbatim, and translated into English. Transcription and translation were conducted by a team of social researchers fluent in all 3 languages with extensive transcription experience. The tapes were destroyed after transcription and transcripts had no identifying information. Data for both activities were collected in June and July 2019. Results were presented to the CHWs and community health focal persons during a community meeting upon completion of data collection and analysis.

### Ethical considerations

Ethical approval for this study was granted by Maseno University Ethical Review Board and the University of Michigan Institutional Review Board. Written informed consent was obtained from all in-depth interview participants prior to the start of the interview. Consent forms were available in English, Kiswahili, or Dholuo and participants chose their language of preference. Each participant signed 2 consent forms, 1 was kept by the research team and the other was left with the participant. This study was a subproject under the larger CHAMPS platform for which ethical approval is granted by the Kenya Medical Research Institute (KEMRI) Scientific Ethical Research Unit (SERU) and the US CDC.

### Data analysis

Analysis began during data collection, as debriefing and discussion occurred daily between the field interviewers and the first author. At the end of each week and after completion of all interviews, the study team reviewed the transcripts to determine emerging themes and discussed these themes and potential codes.

Transcripts were subsequently further analyzed using thematic inductive coding in Dedoose software (Dedoose version 8.2.14, web application for managing, analyzing, and presenting qualitative and mixed method research data, 2019; SocioCultural Research Consultants, LLC; www.dedoose.com). Due to limited resources, codes were developed by the first author only based on discussions and systematically applied to transcripts. Themes were developed inductively from codes, participant responses, and researcher discussions. Themes were then used to develop a conceptual framework. The development of the conceptual framework was guided by the data and research team discussions.

## Results

**Table 2** shows the sociodemographic characteristics of the 20 mothers who participated in in-depth interviews. Most mothers were between 20 and 30 years old, had 1–3 children, were married, and had less than a secondary education. All mothers identified as Christian.

**TABLE 2:** Demographic characteristics of mothers interviewed through the in-depth interview^1^

Characteristic	Number interviewed
Age of child	
6–8 mo	6
9–11 mo	4
12–18 mo	8
19–23 mo	2
Age of mother	
20–25 y	10
26–30 y	8
31–35 y	2
Marital status of mother	
Single	2
Married	13
Separated	4
Divorced	1
Parity	
1	5
2	4
3	7
4	2
≥5	2
Religion	
Christian	20
Other	0
Educational level	
Primary education or below	7
Some secondary	6
Completed secondary	5
Some university	2

1 Information was asked directly at the beginning of each interview.

Themes that emerged from the in-depth interviews and focus group discussions were used to develop a framework that describes community, household, and social and cultural factors influencing inadequate feeding practices in the community. Themes and the number of times they were referenced in transcripts are shown in **Table 3**. These themes are discussed in the following sections.

**TABLE 3:** Frequency of themes discussed in interviews and focus group discussions

Theme	Count
Interpersonal relationships	31
Income	74
Employment	66
Water/hygiene	31
Food environment	120
Health care providers	58
Cultural food beliefs	36

### Community and household income and employment opportunities

Participants described income and employment opportunities for women in the community as a foundational concern, connected to every other theme mentioned. Nearly all mothers discussed how their practices do not always align with recommendations and expressed a desire to feed their child a greater variety of foods, feed their child more frequently, or buy a different brand of food products in order to better meet guidelines. Products most frequently discussed were fruits, milk, and brand-name infant cereals. The largest driving factor behind mother's inability to make these changes was cost.

*“…Where I come from the biggest challenge is you may want to give the child a balanced diet, carbohydrates, vitamins, proteins, and fats, but what is accessible is carbohydrates. They are not so expensive. Proteins are expensive so you find that this is why we feed children one type of food.” —*Mother, focus group discussion

Mothers and CHWs drew a direct connection between the lack of opportunities for renumeration outside of the home for women in the community and household income. Women who were able to find work outside the home had to leave their child at home or find a caregiver. Either option was seen by mothers as negatively impacting their child's nutrition, as a caregiver may not be properly educated on how or what to feed the child, resulting in early termination of breastfeeding and an increase in the quantity of cheap, quick foods in their child's diet. Financial and employment barriers were also perceived as directly impacting the time a mother had to focus on feeding the children. Mothers reported being busy with multiple children, informal jobs, and/or significant household duties. These also impacted the time available to engage with the formal and informal health sector about their child's health and nutrition.

*“The challenge we have is sometimes we cannot get to those areas… let talk of clinics, you may wish to go early so that you get the teachings, but you do not have fare for transport. Even for those who visit [CHWs] you may wish to sit down with them but there is no time because you have to go to work.”* —Mother, in-depth interview

### Community and household water and sanitation environment

Mothers described the impact their household and community environments had on their ability to properly feed their child. In interviews, mothers often referenced the need to boil water and pay careful attention to the cleanliness of the child's food. In focus group discussions, both mothers and CHWs felt that the lack of community-level water and sanitation infrastructure was a barrier to complete cleanliness and proper hygiene within the home. This lack of cleanliness was, in turn, directly related to less hygienic food preparation and a lack of clean spaces for the child to eat. In addition, lack of clean water also meant fewer clean homes in general and that pests and animals could get into the food.

*“…In the area where I live most people do not have water, piped water. You will find that at times they draw water from the well. Water from the well is not clean even for washing hands. You can cook with it after boiling it, however you can't drink it. So, you find that it causes diseases just as my colleagues have said—you will find that a child does not develop well*.*”* —CHW, focus group discussion

### Community and household food environment

The community and household food environment also impacted mothers’ feeding practices. Women talked about food availability in the community, mentioning that foods they wanted, such as bananas, fish, and squash/pumpkin, were often unavailable in the local markets. Street foods were frequently described as high in fat and bad for children but were also regarded as readily available and cheap, therefore occasionally fed to children. Food availability at the household level was dictated by the affordability of the food within the community food environment. The food actually purchased and fed to the child was driven by different factors in different households. In some, it depended on how much money mothers were given by the child's father to buy food, while in others the father himself bought the food for the family. Mothers noted a clear effect on child feeding depending on who in the family had opportunities to work outside the home for renumeration and therefore held the financial power in the family.

*“The challenge we see when feeding the children is for us to get a balanced diet. You may want your child to eat something but for you to get it in terms of money that you want to buy it with it is a challenge. Mostly if only the father is working, and the mother is not working. The mother may want something, but she is not in a position and this may bring conflicts and sometimes children may end up not eating.”* —Mother, focus group discussion

### Social and cultural factors

#### Relationship with health care providers

Mothers reported a sense of trust in the formal health care system, often turning to doctors, nurses, and nutritionists for questions regarding feeding. However, some noted the inconsistencies among staff members at the clinics, which impacted the quality and quantity of nutritional education mothers received in clinics.

*“* *Sometimes you go to the clinic or go to the hospital, and some of the nurses they only do what is necessary, as in if the child is sick, that is what they are concerned about, but there are other nurses seeing the child will advise you, do this, cook for him this, so not every time you will get the information on how to feed the child*.*”* —Mother, in-depth interview

CHWs were also mentioned as sources of information regarding child feeding. Mothers generally felt that CHWs felt they could support them better by providing financial or in-kind resources so they could follow best practices. Currently, only vitamin A supplementation is provided to the community biannually. While the information provided and the visits themselves appeared to be helpful, changes in behavior in response were difficult due to constraints.

*“They [CHWs] may teach you then sometimes it becomes difficult to find those things [foods] because of scarce resources. If there could be a way in which they can support us it can be good.”* —Mother, in-depth interview

CHWs reported that they often received questions from mothers about feeding and nutrition and felt confident in their ability to provide accurate and timely nutrition-related education. They echoed the sentiment that mothers are often frustrated with the lack of resources to follow the recommendations they provide.

*“When you discuss a child's feeding with a parent, they will ask you ‘You are here teaching us but what have you brought for us? Bring us something if it is the government who has sent you, let them give us food to feed the children. Whatever you are saying is good however we will not do them.’”* —CHW, focus group discussion

#### Interpersonal relationships

Other personal relationships were also important sources of information for mothers, particularly the child's father, the mother's mother or mother-in-law, and neighbors. Mothers, mothers-in-law, and other female family members were sources of advice on child feeding and were often the first ones to teach the new mothers how to feed her child. Many also talked about friends and neighbors as important sources of support and advice.

*“I can ask the neighbor because she is older than me, I can ask her what to feed the child. I used to mill porridge flour and mixed cassava, sorghum, millet and groundnuts and I saw that the baby had diarrhea.… I asked my neighbor what may be causing the diarrhea.… She said maybe it is the cassava and it has compelled me to stop giving cassava and buy toto afya [local trade name for baby porridge].”* —Mother, in-depth interview

#### Cultural food beliefs

Mothers demonstrated a strong knowledge of recommended feeding practices, which was evident as they often expressed a desire to feed their children more fruits and vegetables and talked about the guidelines to exclusively breastfeed until the child reaches 6 mo. However, cultural beliefs contrary to international guidelines were also evident. Such beliefs included children should not eat meat, fish, and sweet potato due to the child's inability to chew and digest these foods. Mothers also described a common belief that eggs caused developmental delays and chose not to feed these to children.

*“… you find that people say that if a child eats too many eggs then they will not be able to speak. This can make someone not give a child eggs as much. There is nutritional value in eggs. So, what people say may discourage you from giving a child a certain type of food or cook it in the house.”* —Mother, focus group discussion

Focus groups revealed that it was common across the community for children to be given food other than breast milk before 6 months of age, especially male children, despite extensive roll-out of breastfeeding-support activities through the Baby Friendly Community Initiative.

*“I am saying other people start giving them porridge and milk at 2 months they say that they do not have milk or they cannot produce milk or the child is often hungry so breast milk is not enough.”* —Mother, focus group discussion

Mothers reported greater difficulties in breastfeeding male children. These included the inability to produce enough milk to satisfy the baby, headaches, and dizziness.

*“My firstborn, a girl I breastfed up to 6 months and I did not have any issues like she was breastfeeding a lot but with a boy this time round I see a difference the boy breastfeeds so much you feel dizzy. I see there is a difference I see that the boy breastfeeds a lot more than the girl. I am beginning to wonder if I will reach 6 months like the girl.”* —Mother, focus group discussion

## Discussion

**FIGURE 1:**
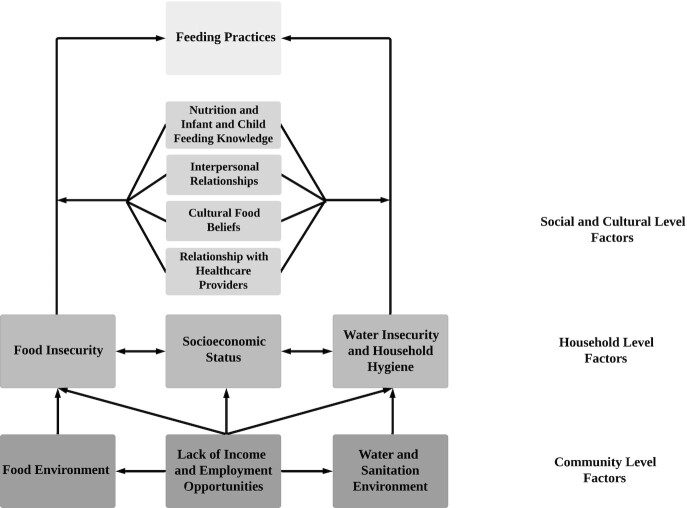
Conceptual framework of mothers’ perceptions of factors influencing complementary feeding practices in a Manyatta urban informal settlement in Kisumu city, western Kenya.

Mothers in this study offer a broad picture of how community, household, and individual factors interact with their nutritional knowledge to result in continued inadequate feeding practices. **Figure 1** demonstrates the pathways through which these different factors interact to shape feeding practices. Underlying mothers’ conceptualization of these factors were personal and community conditions of poverty, including low incomes and limited formal employment opportunities for women. Mothers further demonstrated the pathways connecting poverty and inadequate feeding by recognizing that poverty results in community-level environments that preclude proper hygiene and feeding, which then produce similar household-level environments. Interpersonal relationships between the mothers and their family and friends, cultural beliefs, and interactions with the formal and informal health care sector, as well as the mother's own nutrition and feeding knowledge, were modifiers of the connection between the community- and household-level factors and the inadequate feeding practices.

Mothers in Manyatta have been exposed to a variety of programs aimed to create behavior change around infant and young child feeding through education (25–27, 36). Our results reinforce findings from other settings that these programs have been successful insofar as mothers demonstrated comprehension of their messages. However, mothers’ actualization of such messaging is largely shaped by external factors outside of their control. For example, mothers noted that without enabling water, sanitation, and food environments, they cannot meet recommendations despite their knowledge of such guidelines. And, even with a more enabling environment, negative feeding practices might persist if individual- and household-level poverty is not addressed. For example, if the availability of healthy foods in neighborhoods increases, it is unlikely that feeding practices will improve if such foods remain financially inaccessible and mothers have limited opportunities for income generation.

This understanding underscores the extent to which researchers and mothers alike see systemic change, particularly around conditions of poverty and their associated drivers, as imperative to progress in complementary feeding practices and associated nutritional outcomes. Poverty and poor standards of living have been shown to be some of the greatest factors underlying a family's ability to feed their child appropriately in Nairobi informal settlements (35, 37). Mothers offer a unique and innovative perspective in the role water plays in their feeding practices, the inclusion of which has been increasingly called for in the nutrition literature by researchers (38). Several recent papers suggest that household water security and food security are related, with increased water insecurity leading to increased food insecurity, just as the mothers and CHWs describe in this context (38, 39). Our results also reinforce previous findings that support from family members is beneficial in adhering to feeding recommendations and that many mothers turn to family and friends for help in child feeding (15, 40). As in other studies, mothers in Manyatta expressed trust in the formal health care system (22, 35). However, the relationships between mothers and CHWs were somewhat unsteady as mothers felt frustrated with the lack of available resources to adhere to their nutritional messaging.

Our results imply some recommendations for intervention and further research. Interventions beyond education are necessary, as mothers’ knowledge is not sufficient to fully address the inadequate feeding practices that contribute to malnutrition, as stated by the mothers themselves. Investment in improved water and sanitation infrastructure may be an important target in this setting. Programs that aim to provide improved water and hygiene and support handwashing along with nutrition interventions could have beneficial effects on the child's growth and development, not only through the direct reduction in pathogen exposure but also potentially through indirect improvements in complementary feeding (41, 42). The role of CHWs in the Kenyan health care system has grown and CHWs are able to navigate the community context differently than those from the formal health care system (43). While CHWs are an important asset in modifying the relation between community factors and feeding practices, support for CHWs may erode if their messaging is not perceived to be feasible. CHW programs have been shown to be successful in other contexts, but challenges regarding frustrations with lack of resources are often present (44–46). CHW programs in conditions of high poverty, as in this setting, may be most beneficial when combined with provision of resources or other similar social protection programs (44). Future programming should not only emphasize inclusion of family members, as has been shown elsewhere, but should also work to include the community as a whole to improve efficacy of such programs, given that friends and neighbors are often sources of information. Such community inclusion could also potentially impact other community-level factors discussed here. Programs to address poverty and to support healthy community environments are critical to improved feeding practices in this context and should accompany activities aimed at increasing maternal nutrition and feeding knowledge. The role that cash transfer or food bank programs, employment opportunities for women, or broader social protection policies may play in indirectly supporting infant and young child feeding practices is an important avenue for future research (47–49).

### Strengths and limitations

Our qualitative study allows for mothers’ and CHWs’ perspectives to be explored in order to create a more complete picture of factors influencing infant and young child feeding practices. However, this study is not without limitations. We did not collect demographic information on participants in the focus groups nor did we collect data on the number of children and adults living in each household interviewed. We also did not examine the perspectives of other important community members, such as fathers, other family members, and formal health care providers, who are also regarded as trusted sources of information and could provide a fuller picture of the infant and young child nutrition environment. Some of the authors are directly involved in the design and implementation of public health programs in this community, but none of these authors led the data collection or analysis, reducing the risk of bias. The sampling method utilized may have excluded mothers representing certain perspectives, but purposive sampling is often used in qualitative work and selection was made by CHWs rather than the study team. Finally, due to resource constraints, data analysis was only conducted by 1 author.

### Conclusions

While mothers living in a Manyatta urban informal settlement have received significant education about complementary feeding, they struggle to act on such knowledge due to the broader systems and conditions in which they live. Examining the critical perspective of mothers themselves, as they attempt to translate recommendations into reality, emphasizes the need for interventions that move beyond individual behavior change and toward systemic transformation.

## Notes

This study was funded by fellowships from the University of Michigan Office of Global Public Health and the University of Michigan Center for the Education of Women.
